# Analyzing Multi-locus Plant Barcoding Datasets with a Composition Vector Method Based on Adjustable Weighted Distance

**DOI:** 10.1371/journal.pone.0042154

**Published:** 2012-07-27

**Authors:** Chi Pang Li, Zu Guo Yu, Guo Sheng Han, Ka Hou Chu

**Affiliations:** 1 School of Life Sciences, The Chinese University of Hong Kong, Shatin, Hong Kong SAR, China; 2 School of Mathematics and Computational Science, Xiangtan University, Hunan, China; 3 School of Mathematical Sciences, Queensland University of Technology, Brisbane, Australia; Auburn University, United States of America

## Abstract

**Background:**

The composition vector (CV) method has been proved to be a reliable and fast alignment-free method to analyze large COI barcoding data. In this study, we modify this method for analyzing multi-gene datasets for plant DNA barcoding. The modified method includes an adjustable-weighted algorithm for the vector distance according to the ratio in sequence length of the candidate genes for each pair of taxa.

**Methodology/Principal Findings:**

Three datasets, *matK*+*rbcL* dataset with 2,083 sequences, *matK*+*rbcL* dataset with 397 sequences and *matK*+*rbcL*+*trnH-psbA* dataset with 397 sequences, were tested. We showed that the success rates of grouping sequences at the genus/species level based on this modified CV approach are always higher than those based on the traditional K2P/NJ method. For the *matK*+*rbcL* datasets, the modified CV approach outperformed the K2P-NJ approach by 7.9% in both the 2,083-sequence and 397-sequence datasets, and for the *matK*+*rbcL*+*trnH-psbA* dataset, the CV approach outperformed the traditional approach by 16.7%.

**Conclusions:**

We conclude that the modified CV approach is an efficient method for analyzing large multi-gene datasets for plant DNA barcoding. Source code, implemented in C++ and supported on MS Windows, is freely available for download at http://math.xtu.edu.cn/myphp/math/research/source/Barcode_source_codes.zip.

## Introduction

The mitochondrial cytochrome *c* oxidase subunit I (COI) has been proposed as the “DNA barcode” region for species identification in the animal kingdom [1], [2]. Since then, a variety of animal groups, such as insects, fishes, birds and amphibians [3]–[7] have already been successfully barcoded with high rates of species discrimination (>90%) [8]. However, the relatively low nucleotide substitution rate in the plant mitochondrial genomes significantly reduces the species discrimination power of COI in plants [9]. Furthermore, with the high structural reorganization in plant genomes [9], searching for a single global DNA barcoding marker, analogous to COI in animals, for plants has been extremely difficult. As a result, the Plant Working Group of the Consortium for the Barcode of Life (CBOL) proposed to use two coding genes, *matK* and *rbcL*, in the plastid genome as the core DNA barcoding markers to discriminate terrestrial plant species [10]. Yet the species discriminatory power of this *matK*+*rbcL* combination was only about 70%, which is much lower than the success rates of species discrimination reported in animals [11]. Kress et al. [9] suggested that the *trnH-psbA* spacer region should be added as the third core DNA barcoding marker because this three-locus marker could provide a “better estimate of species identity”, and it is very easy to be amplified by PCR across terrestrial plants using a pair of universal primers. This *matK*+*rbcL*+*trnH-psbA* combination, with two coding regions plus one non-coding region, seems to be the most promising DNA barcoding strategy to discriminate terrestrial plant species up to date.

Multiple alignment of large single-locus DNA barcoding datasets is always time consuming and requires high computing power [12], and the Barcode of Life Data Systems (BOLD) has to divide the large barcode dataset into several “sub-projects” with a size limit of 5,000 specimens each for analysis [13]. The latest multi-locus approach, either the *matK*+*rbcL* or *matK*+*rbcL*+*trnH-psbA* combination, suggested for plant DNA barcoding would further challenge the computing capacity of alignment algorithms. In particular, including the non-coding region *trnH-psbA* as one of the core DNA barcoding markers is problematic, as base insertions and deletions (indels) are commonly found. In fact, inverted repeats have been reported in *trnH-psbA* among gymnosperms [14]. Such indels and repeats could make sequence alignment ambiguous because assigning gaps to DNA sequences is highly subjective [15], and there is no consensus on what defines a “good” or “best” multiple alignment [16]. Kress et al. [9] were also concerned about the fallacy in aligning *trnH-psbA* sequences from highly divergent taxa, and suggested that the non-coding sequences should be aligned in nested groups before combining with the coding sequences for global alignment. This multi-step multiple alignment protocol would make the already problematic alignment process even more troublesome and time-consuming, especially for large DNA barcoding datasets. Moreover, this process often has to be repeated whenever a new sequence is added to a dataset. The ultimate solution is to develop a fast and effective alignment-free analytical method to handle the multi-locus datasets for plant DNA barcoding projects.

Our previous studies [12], [17] show that the composition vector (CV) method is a fast and reliable alignment-free approach for analyzing large sequence datasets, including those of non-protein-coding genes, such as rRNA. Briefly, the CV method is a simple correlation analysis based on composition vectors derived from either DNA or amino acid sequences. First, the CV of each sequence is obtained by determining the frequencies of short DNA strings, and then the pairwise distance between the CVs is calculated. Finally, a distance tree using the neighbor-joining (NJ) method is generated based on the distance matrix. So far, only single-locus datasets have been tested with the CV method in our studies. To handle multi-locus datasets from plant DNA barcoding projects, the CV method has to be modified. In this study, an adjustable-weighted algorithm for the vector distance according to the pairwise ratio in sequence length of the candidate genes is incorporated. Accordingly, the distance matrix for each gene segment generated by the CV method is weighted before combining for further analysis. Three datasets, *matK*+*rbcL* dataset with 2,083 sequences, *matK*+*rbcL* dataset with 397 sequences and *matK*+*rbcL*+*trnH-psbA* dataset with 397 sequences, were tested in the present study. The two 397-sequence datasets were from the CBOL Plant Working Group [10], while the 2,083-sequence dataset was largely based on Little's dataset [18]. These datasets were chosen because they include the most promising DNA markers proposed for plant barcoding, and they also contain the largest number of plant DNA sequences published so far. The objective of this study was to evaluate how well the modified CV method could handle the multi-locus DNA barcoding datasets for plants, and the results showed that the success rates of grouping sequences at the genus/species level based on the modified CV approach are always higher than those based on the traditional analytical method.

## Methods

The 397-sequence dataset analyzed in the present study contains three loci, *matK*, *rbcL* and *trnH-psbA*, from 99 genera and 220 species, and is available in CBOL Plant Working Group [10]. The 2,083-sequence dataset, which is largely based on the dataset of Little [18], was kindly provided by Dr. Damon P. Little of the New York Botanical Garden, and it contains two loci, *rbcL* and *matK*, from 977 genera and 1,737 species.

The basic principle of the composition vector (CV) method was fully described previously [19]–[23], including its application in DNA barcoding [12], [17], and phylogeny of chloroplasts [22], [24], large dsDNA viruses [25], [26] and fungi [27]. Briefly, for a sequence of a gene of length *L*, the frequency of the appearance of oligonucleotide strings of a fixed length *K* is calculated. The total number of possible types of such strings is 4*^K^* and the total number of *K*-strings is (*L*−*K*+1). The frequency of each of the *K*-strings in a given DNA sequence is determined by sliding through the sequence, shifting one nucleotide position at a time. The observed frequency *p*(α_1_α_2_… α*_K_*) of a *K*-string α_1_α_2_… α*_K_* is *n*(α_1_α_2_… α*_K_*)/(*L*−*K*+1), where *n*(α_1_α_2_… α*_K_*) is the number of times that α_1_α_2_… α*_K_* appeared in this sequence. For a certain *K*, we put the frequencies of all possible *K*-strings in a fixed order to obtain a composition vector of dimension 4*^K^* for each sequence. The correlation *C*(*A,B*) between two sequences *A* and *B* is determined by taking the projection of one vector on another, and the distance between the two is defined as *D* = (1−*C*)/2. In the modification to handle multi-locus dataset, the pairwise distance from each gene is weighted according to their sequence length before combining distances for subsequent analysis. For instance, in the case of two genes, if we use 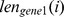 to denote the length of *gene*_1_ in species “*i*”, and so on, and define


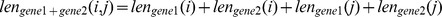


Then we define the weights of *gene*_1_ and *gene*_2_ in a pair of taxa *i* and *j* as


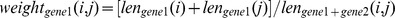



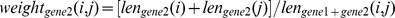


It can be seen that the weights of two genes in a pair of taxa *i* and *j* are independent on the string length *K* (meaning that for any value of *K*, the weights are the same). If we consider string length *K*_1_ for *gene*_1_ and *K*_2_ for *gene*_2_ and have obtained the CV distances 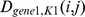 and 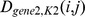 as in our previous studies [12], [17], we define the weighted CV distance based on *gene*_1_ and *gene*_2_ between species *i* and species *j* as


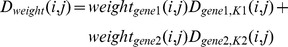


**Figure 1:**
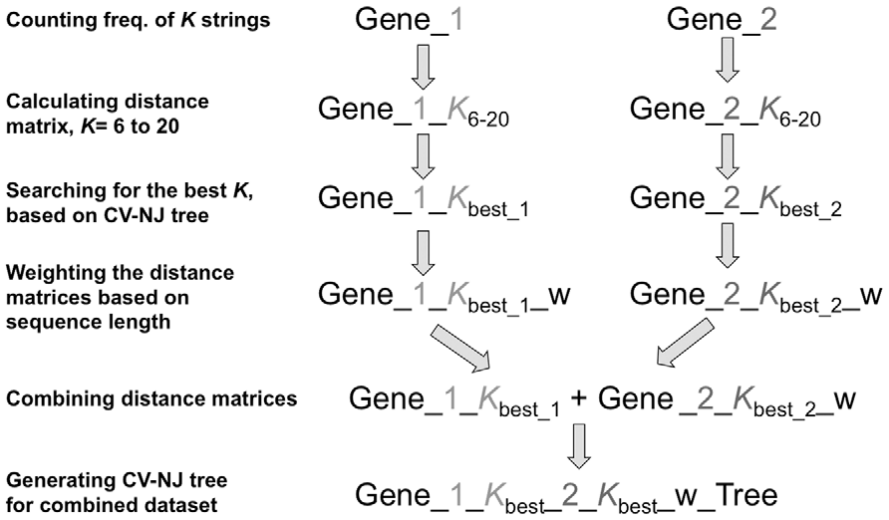
Workflow of the modified composition vector method for analyzing multi-locus datasets.

By this equation, the distance matrices from each gene marker based on different string length *K* can be combined together, and the neighbor-joining (NJ) [28] tree construction based on the weighted, combined distance matrix from all selected genes or loci, can then be performed by Phylip 3.66 [29]. A summary chart on the workflow of the newly modified CV method is shown in Figure 1.

To determine the best length of string (*K*) used in the CV analysis, the distance matrices for individual gene from *K* = 6 to 20 were generated. Then, the best *K* for individual gene was determined according to the success rate of grouping sequences correctly at the genus/species level (see below). Finally, the distance matrix, *D*(*i, j*), generated from the best *K* for each selected gene was used in the combined analysis. The CV/NJ tree of each dataset generated from the combined distance matrices was then compared to the corresponding K2P/NJ trees constructed using the traditional methodology with sequence alignment constructed as follows. First, the DNA sequences for each gene segment were aligned using MUSCLE [30], and the aligned datasets were combined for analysis. Finally, the NJ tree was constructed using Mega 5 [31] based on Kimura 2-parameter (K2P) distance model [32].

To compare the grouping effectiveness of the CV/NJ tree and K2P/NJ tree from each of the three datasets, we estimated the percentage of sequences that could be successfully grouped at the genus/species level as well as the percentage of species with multiple sequences that could be successfully grouped (Table 1). The first percentage refers to grouping success of sequences at the genus/species level, i.e., all the sequences from species within the same genus or those from multiple individuals of a given species have to be clustered together as a group without including any sequence from other genera or species. The second percentage value refers to grouping success of species with multiple sequences where the sequences from a given species have to be grouped together without including any sequence from other species.

**Table 1:** The grouping effectiveness of the K2P/NJ and CV/NJ methods for the three plant barcoding datasets.

	% Success in grouping sequences to species/genus			% Success in grouping species with multiple sequences		
Dataset	N_1_	K2P/NJ method	CV/NJ method	N_2_	K2P/NJ method	CV/NJ method
397-sequence (*matK*+*rbcL*)	383	70.7%	77.2%	102	62.7%	70.6%
397-sequence (*matK*+*rbcL*+*trnH-psbA*)	383	68.1%	81.7%	102	60.8%	77.5%
2,083-sequence (*matK*+*rbcL*)	1,319	55.3%	55.4%	204	44.6%	52.5%

N_1_ – total number of sequences from genera with multiple species or species with multiple individuals. N_2_ – total number of species with multiple sequences.

## Results

For the 397-sequence dataset [10], the best *K* value of 14 was selected for both the *matK* and *rbcL* regions, and *K* of 8 was selected for the *trnH-psbA* region. In the CV/NJ trees, the success rates of grouping sequences at the genus/species level for the combinations of *matK*+*rbcL* and *matK*+*rbcL*+*trnH-psbA* were 77.2% and 81.7%, respectively (Table 1). The corresponding values for the K2P/NJ trees were 70.7% and 68.1%. In terms of the success rates of grouping species with multiple sequences in the CV/NJ trees, the values were 70.6% and 77.5%. For the K2P/NJ tree, they were 62.7% and 60.8%. In the study by the CBOL Plant Working Group [10], the success rates of species discrimination, which were restricted to species where multiple individuals were sampled from congeneric species with the discrimination considered successful if the minimum uncorrected interspecific *p*-distance is larger than the maximum intraspecific distance, are 71.6% for both the *matK*+*rbcL* and *matK*+*rbcL*+*trnH-psbA* combinations.

For the 2,083-sequence dataset, the best *K* value of 14 was selected for both the *matK* and *rbcL* regions. The success rates of grouping sequences at genus/species level were 55.4% and 55.3% for CV/NJ and K2P/NJ, respectively (Table 1). In terms of the success rate of grouping species with multiple sequences, the corresponding values were 52.5% and 44.6%.

## Discussion

The CV method has been proved to be an efficient algorithm to analyze large single-locus DNA barcoding datasets [12], [17]. In the present study, the CV method has been modified for handling multi-locus datasets for plant DNA barcoding, and it achieves the highest grouping success in the *matK+rbcL*+*trnH-psbA* dataset. Therefore, we believe that our method is suitable for analyzing multi-locus barcoding datasets in plants. In fact, our analysis shows that the CV method always outperforms the traditional method, i.e., K2P/NJ, in grouping sequences to genus/species in both the *matK+rbcL* and *matK+rbcL*+*trnH-psbA* datasets tested in this study. For the 397-sequence dataset, both *matK+rbcL* and *matK+rbcL*+*trnH-psbA* datasets were tested. For the *matK+rbcL* combination, the success rate of grouping sequences to genus/species with the CV/NJ method is higher than that using the K2P/NJ method by 6.5%. With the addition of the *trnH-psbA* spacer region, the success rate using the CV/NJ method increases by 4.5% to 81.7%. Unlike the result from the CV/NJ method, adding the *trnH-psbA* spacer region in the K2P/NJ method does not improve the grouping effectiveness, which actually decreases by 2.6%. By using the traditional methods which are based either on direct measurement of pairwise distance from global alignments [10] or the K2P/NJ method used in the present study, adding one more gene, i.e., the *trnH-psbA* spacer, does not enhance the discriminatory or grouping power. For instance, a success rate of 71.6% was reported for both the *matK+rbcL* and *matK+rbcL*+*trnH-psbA* combinations [10]. Moreover, the average species discriminatory power in plant barcoding was only about 70% for all possible seven-locus combinations [10]. The saturation of discriminatory power found among the different marker combinations may be caused by the poorly aligned sequences from the non-protein-coding regions. In contrast, since the CV/NJ method does not require DNA sequence alignment, adding more gene regions, especially non-protein-coding regions, would enhance the grouping power as shown in the present study.

For the 2,083-sequence dataset based on the *matK*+*rbcL* combination, as in the 397-sequence dataset, the success rate of grouping sequences in CV/NJ method is always higher than that found in the K2P/NJ method. However, the grouping effectiveness is significantly lower (by about 20%) in the 2,083-sequence dataset than in the 397-sequence dataset. The weak grouping effectiveness found in the 2,083-sequence dataset may be caused by the presence of a large number of single taxon sequences, i.e., those from species or genus that have no other sequences in the dataset. There were 764 single taxon sequences found in this dataset, yet many of these sequences were found to cluster within groups consisting of species with multiple individuals, or multiple congeneric species, thus resulting in low grouping effectiveness. The aberrant phylogenetic positions of some of these problematic single taxon sequences found in the CV/NJ and K2P/NJ trees might result from poor sequence data or DNA contamination from other species. Further, we could not exclude the possibility that some of these single taxon sequences are the result of misidentification of species, such that some of the sequences with wrong species names were actually assigned to their correct species or congeneric group based on DNA barcoding. Further, it should be noted that the dataset of Little [18] available for public download actually contains 1,745, not 2,083 sequences. The smaller dataset exclude 338 sequences from multiple individuals of the same species. Thus, this dataset could not be used in the present study as we aim to analyze for grouping effectiveness at the species level. It should be noted that the grouping success reported in the present study could not be compared directly with the success species “discriminatory rates” based on global alignment method [10], [18] because, in the latter method, the correct species identification relies on the “barcode gap” that is based on inter- and intra-specific genetic distances. As a result, failure in identifying a query sequence would not affect the species discriminatory result on the other queries since the method is based on pairwise matching algorithm. Our CV/NJ method, however, is a tree-based method, and any sequences that do not match the others from the same taxon lead to failure in grouping. Thus in our study, the grouping success from the K2P/NJ method and CV/NJ method reported in the present study can be compared directly, and clearly the CV/NJ method always outperforms the K2P/NJ method.

In alignment-free methods of sequence analysis based on DNA strings, a critical factor is the length of the string, *K*, for analysis. In our previous CV barcoding studies [12], [17], we followed the method of Pevzner [33] to determine the best *K* value for the CV analysis. In preliminary studies of the present work, we obtained the best *K* value using Pevzner's method [33] for the three datasets, but the grouping effectiveness of these CV/NJ trees is low. For instance, the best *K* value is 9 for the *rbcL* dataset according to this method yet the grouping effectiveness at the genus/species level of the tree based on this value is only 64.4%, which is substantially lower than 68.2% for the tree with a *K* value of 14. Thus Pevzner (2000)'s method [33] was not adopted in estimating the best *K* value in this study. The reason why Pevzner (2000)'s method [33] failed to estimate the best *K* is yet to be explored. In order to search for the best *K* value, CV/NJ trees from each individual gene from *K* = 6 to 20 were generated in the present study, and the *K* value with the highest grouping effectiveness at the genus/species level was selected as the value for that particular gene region in the combined analysis. Although this method was assumed to be the best approach in determining the best *K*, it was very time-consuming. One of the advantages of using the CV method is its fast analytical speed, so that the slow rate-determining step of searching for the best *K* value is undesirable. We suggest using a preset *K* value for a particular gene region until we develop an automated tool for selecting the best *K* value. In fact, while the best *K* value may vary among different gene markers (i.e., 14 for *rbcL* and *matK* and 8 for *trnH-psbA*), it appears that it remains unchanged among different datasets of the same gene marker, since the best *K* value of 14 was found in *rbcL* or *matK* for both the 397-sequence and 2,083-sequence datasets. Thus we believe this *K* value can be adopted to analyze any other datasets of these two genes. However, if a new gene region, other than *matK*, *rbcL* and *trnH-psbA*, is added to the plant DNA barcode combination, the best *K* value for that particular gene has to be determined by searching for the best CV/NJ tree among those generated from different *K* values.

The major modification of our modified CV method is to incorporate an adjustable-weighted algorithm for the vector distance according to the ratio of sequence length found between a pair of taxa in the candidate genes. In fact, our preliminary studies show that the CV/NJ trees with the weighted distance could always provide a higher grouping effectiveness than the CV/NJ trees without using the weighted distance. For instance, the grouping effectiveness of sequences to genus/species for the *matK*+*rbcL* dataset using the weighted distance was 0.7% higher than the corresponding value without using the weighted distance. This distance weighting process is critical, especially when the length variation among the selected genes in a multi-locus dataset is high. Besides the variable lengths found in different gene regions, the different nucleotide substitution rates among the selected gene regions would also affect the discriminatory power in the combined analysis. This difference should be taken into account for further improvement of analyzing barcoding dataset using the CV approach. In the present study, we demonstrated the power of the CV method in analyzing large DNA barcode datasets of multiple gene regions, regardless of the type of gene markers used. In the tested datasets, the CV/NJ method always gives higher grouping success (in terms of sequences or species) than the conventional method of K2P/NJ. To conclude, the modified CV/NJ method can be adopted as an effective and fast tree construction algorithm in analyzing multi-locus DNA barcode datasets.
